# RNAi Silencing of the HaHMG-CoA Reductase Gene Inhibits Oviposition in the *Helicoverpa armigera* Cotton Bollworm

**DOI:** 10.1371/journal.pone.0067732

**Published:** 2013-07-02

**Authors:** Zhijian Wang, Yongcheng Dong, Nicolas Desneux, Changying Niu

**Affiliations:** 1 Hubei Key Laboratory of Insect Resource Application and Sustainable Pest Control, Plant Science & Technology College, Huazhong Agricultural University, Wuhan, China; 2 French National Institute for Agricultural Research (INRA), Sophia-Antipolis, France; Centers for Disease Control and Prevention, United States of America

## Abstract

RNA interference (RNAi) has considerable promise for developing novel pest control techniques, especially because of the threat of the development of resistance against current strategies. For this purpose, the key is to select pest control genes with the greatest potential for developing effective pest control treatments. The present study demonstrated that the 3-hydroxy-3-methylglutaryl coenzyme A reductase (HMG-CoA reductase; HMGR) gene is a potential target for insect control using RNAi. HMGR is a key enzyme in the mevalonate pathway in insects. A complete cDNA encoding full length HMGR (encoding an 837-aa protein) was cloned from *Helicoverpa armigera* (Lepidoptera: Noctuidae). The HaHMGR (*H. armigera* HMGR) knockdown using systemic RNAi *in vivo* inhibited the fecundity of the females, effectively inhibited ovipostion, and significantly reduced vitellogenin (Vg) mRNA levels. Moreover, the oviposition rate of the female moths was reduced by 98% by silencing HaHMGR compared to the control groups. One-pair experiments showed that both the proportions of valid mating and fecundity were zero. Furthermore, the HaHMGR-silenced females failed to lay eggs (approximate 99% decrease in oviposition) in the semi-field cage performance. The present study demonstrated the potential implications for developing novel pest management strategies using HaHMGR RNAi in the control of *H. armigera* and other insect pests.

## Introduction

RNA interference (RNAi), which was first characterized in *Caenorhabditis elegans* [1], has been developed as an effective gene-silencing tool in a wide variety of organisms [2]. Double-stranded RNA (dsRNA)-mediated RNAi has emerged as one of the most powerful strategies for the rapid analysis of gene function and has considerable potential for the development of applications for insect pest control [3], [4], [5], [6]. In recent years, increasing amounts of genome and transcriptome sequencing data of important pest species have been made available online. Genetic function determination and novel control method developments by RNA interference could be reasonably combined for the integrated pest management (IPM) of important insect pests in the near future to address the continuous threat of resistance development against current pest management techniques [7], [8]. The achievement of this objective can be accelerated by targeting pest control genes. For example, the key candidate genes in the pathways that regulate insect development and reproduction are promising targets for the implementation of pest control using RNAi technology.

HMG-CoA reductase (HMGR) is one of the current candidate genes for this potential application. HMGR is the key regulatory enzyme in the mevalonate pathway, which controls a rate-limiting step in the conversion of HMG-CoA into mevalonate, a precursor for the synthesis of cholesterol in vertebrates [9]. However, insects do not synthesize cholesterol through the mevalonate pathway [10]. Instead, insects produce juvenile hormones (JHs) that regulate development and reproduction in most insect species [11]. Over the past decade, researchers have discovered that HMGR may have various essential roles in the regulation of embryonic development, induction of vitellogenin synthesis and pheromone production in insects. For example, the regulatory role of HMGR has been widely investigated in cockroach species, including *Blattella germanica* and *Diploptera punctata* [12], [13]. Studies have shown that inhibitors of HMGR can prevent the synthesis of vitellogenin [14], [15] and can also reduce the fecundity of *B. germanica*. However, HMGR should not be considered the rate-limiting enzyme for JH synthesis by the corpora allata in *D. punctata* [15], [16]. An inhibitor of HMGR can inhibit sex pheromone biosynthesis in *Bombyx mori* and *Spodoptera litura* [17], [18]. HMGR regulates the maintenance of homeostasis between the *de novo* produced and sequestered intermediates of iridoid metabolism in the leaf beetle [19]. In addition, HMGR can mediate the transfer of origin germ cells in *Drosophila melanogaster* [20], [21]. Other studies using *in situ* hybridization have revealed that HMGR is highly expressed in specialized cells of the male anterior midgut where monoterpenoid aggregation pheromones are synthesized *de novo* [9].

The HMGR gene has been cloned and identified from many insect species across several insect orders, including *Agrotis ipsilon* [22], *B. mori* [23], *Samia cynthia ricini* [24], *B. germanica* [25], *Phaedon cochleariae* [19], *Dendroctonus jeffreyi* [26] and *Ips pini* [27]. In addition, molecular characterizations of HMGR have been accomplished in *D. melanogaster* [28], *B. mori* [23], *A. ipsilon* [22], and *B. germanica* [25], which have initiated gene function research along with potential applications for RNAi.

On the basis of sequence homologies of the genes deposited in GenBank, we recently identified a putative HMGR gene in the cotton bollworm, *H. armigera*, which is one of the major insect pests in the world. Despite the lack of a sequenced genome, the physiology, metabolism and reproduction of *H. armigera* have been studied intensively because of its devastating nature. Here, we examined the role of the HaHMGR gene in the reproduction of this moth using RNAi. Knockdown of the HaHMGR gene by injecting HaHMGR dsRNA into 2-day-old female pupae influenced the mating of the adults and significantly inhibited oviposition. This finding may have important implications for the development of effective pest control against this moth and other insect pests.

## Materials and Methods

### Insects

*H. armigera* were reared on an artificial diet in regulated climatic chambers (27±1°C, RH of 40±10%, and photoperiod of 14L:10D). The adults were maintained in rearing cages (40 cm×30 cm×30 cm) and fed with a 10% (sugar/water) sucrose solution until their use in the experiments.

### Cloning of the HaHMGR and Vg Genes

Fat bodies were dissected from 1-day-old female adults and then immediately frozen in liquid nitrogen. Total RNA was isolated from the fat bodies using Trizol (Invitrogen, Carlsbad, CA). First-strand cDNA synthesis was performed using the RevertAid First Strand cDNA Synthesis Kit (Fermentas, EU) with oligo(dT) primers. Degenerate primers (HMGR-F/HMGR-R) were designed for the amplification of a specific fragment of HMGR (Table 1). PCR amplifications were performed in 25 µl volumes containing 1 µl of primers, 2.5 µl of 10× buffer, 2 µl of each dNTP, 0.15 µl of Ex Taq (TaKaRa, Dalian, China) and 1 µl of cDNA template, and the following thermocycler protocol was used: 35 cycles of 95°C for 30 sec, 59°C for 30 sec, and 72°C for 3 min. Gene-specific primers (HMGR-F3-1, HMGR-F3-2, R3-1 and R3-2) were designed for 3′-rapid amplification of cDNA ends (3′-RACE) (Table 1). The outer PCR protocol consisted of 20 cycles of 95°C for 30 sec, 58°C for 30 sec and 72°C for 1 min. The PCR product was used as the template for the inner primer with the following protocol: 30 cycles at 95°C for 30 sec, 58°C for 30 sec, and 72°C for 1 min. The 5′-RACE reactions were performed using the 5′-Full RACE kit (TaKaRa, Dalian, China). The outer PCR protocol consisted of 20 cycles of 95°C for 30 sec, 55°C for 30 sec, and 72°C for 1 min. The PCR product was used as the template for the inner primer, and the thermocycler conditions were as follows: 30 cycles at 95°C for 30 sec, 58°C for 30 sec, and 72°C for 1 min.

**Table 1:** PCR primers for HaHMGR cDNA cloning from *Helicoverpa armigera*.

Primer set	Primer sequence
Degenerate primer	(HMGR-F) 5′-ATGAAAGTSTGGGGAGCYCACG-3′
	(HMGR-R) 5′-TCACAAGGGCAGCCATTAG-3′
	(VG-F) 5′-GGBAACYGAGCSADCAGCAG-3′
	(VG-R) 5′-CCTGYACTGDTGGMCAGCC-3′
3′-RACE primer	(HMGR-F3-1) 5′-ATTGGCGGGAATAACGCTCACGC-3′
	(HMGR-F3-2) 5′-TGGAGGGACTATCCTAACAGGCC-3′
	(R3-1) 5′-GCTGTCAACGATACGCTACGTAACGGCATGACAGTGTTTTTTTTTTTTTTTTTTTTTTTT-3′
	(R3-2) 5′-CGCTACGTAACGGCATGACAGTG-3′
	(VG-F3-1) 5′- GTGAAATCTGCATCACCACCACCC -3′
	(VG-F3-2)5′- CGGAGAAGGTTACAAGGTCCAAGC -3′
5′-RACE primer	(F-5-1)5′-CATGGCTACATGCTGACAGCCTA-3′
	(F-5-2) 5′-CGCGGATCCACAGCCTACTGATGATCAGTCGATG-3′
	(HMGR-R5-1) 5′- GGGCATCCTTAATACTCGCCAG-3′
	(HMGR-R5-2) 5′-AGCGTACGAAGGTCATTATTACG-3′
	(VG-R5-1)5′- AGCAGACCCTTGAGTAAGTTCTCG -3′,
	(VG-R5-2)5′- GGACTGGGTTCTCCAACTTGG -3′
Quantitative PCR	(QActin-F)5′-TCCAGCCCTCATTCTTGGGTAT-3′
	(QActin-R)5′- CAAGTCCTTACGGATGTCAACA-3′
	(QHMGR-F)5′- TACAGTAGGTGGAGGGAC-3′
	(QHMGR-R)5′- ATCAAGGAGGCTAATCGGG-3′
	(QVG-F)5′-CGGAGACAAGAAACAGAACAC-3′
	(QVG-R)5′-AAGCAATAATGCGGACGAGAAT-3′
RNAi	(T7EGFP-F)5′-TAATACGACTCACTATAGGGAGACCCTGAAGTTCATCTGCACC-3′
	(T7EGFP-R)5′- TAATACGACTCACTATAGGGAGAGTGCTCAGGTAGTGGTTGTC-3′
	(T7HMGR-F)5′-TAATACGACTCACTATAGGGAGATCCCTATGGCTACAACTGAAGG-3′
	(T7HMGR-R)5′-TAATACGACTCACTATAGGGAGACCAGCCGATTTAAGCAC-3′

To clone the *H. armigera* Vg gene, degenerate primers (VG-F and VG-R) were designed (Table 1). PCR amplifications were performed in 25 µl volumes containing 1 µl of primers, 2.5 µl of 10× buffer, 2 µl of each dNTP, 0.15 µl of Ex Taq (TaKaRa, Dalian, China) and 1****µl of cDNA template. The following thermocycler program was used: denaturation at 95°C for 30 sec (2 min for only the first cycle), annealing at 55°C for 30 sec and extension at 72°C for 5 min for 35 cycles. To obtain the complete cDNA sequence of the Vg gene, a new set of gene-specific primers (VG-F3-1, VG-F3-2, VG-F5-1 and VG-F5-2) matching the primers in the 3′- and 5′-Full RACE kit (Takara, Dalian, China) were designed (Table 1). The 3′-RACE outer and inner PCR reactions were carried out with 20 cycles at 95°C for 30 sec, 55°C for 30 sec, and 72°C for 1 min followed by 30 cycles at 95°C for 30 sec, 60°C for 30 sec, and 72°C for 1 min. The 5′-RACE outer and inner PCR amplification conditions were the same as the 3′-RACE outer and inner protocols. All clones were sequenced by Invitrogen (Shanghai, China).

### Sequence Alignments and Comparisons

To compare the identified HaHMGR sequence (GenBank accession no. GU584103) to other insect species, we used the previously published HMGR amino acid sequences of the following species: *A. aegypti* (XP_001659923), *A. ipsilon* (O76819), *A. grandis* (AF162705), *B. mori* (NM_001099828), *C. quinquefasciatus* (XM_001845655), *D. jeffreyi* (AF159136), *D. melanogaster* (NM_170089), *I. pini* (AF304440), *I. paraconfusus* (AF071750), *Apis mellifera* (BI503396), *Bombus impatiens* (XM_00349205), *I. confuses* (FJ536869), *Chrysomela populi* (EF134409), *P. cochleariae* (EF134407), *Gastrophysa viridula* (EF134408), *S. cynthia ricini* (DQ465407), and *B. germanica* (P54960). Sequence alignments were carried out with MEGA4 Molecular Evolutionary Genetics Analysis Software Version 4.0 (Tokyo, Japan).

### HaHMGR and Vg Quantitative real-time PCR (qPCR)

To determine the appropriate age for initiating RNAi silencing of the target gene, we used qPCR to investigate the relative expression of HaHMGR in female pupae from day 1 to day 9. The same method was also used to assess the expression levels of Vg in female pupae from day 1 to day 9.

Total RNA was isolated using Trizol (Invitrogen, Carlsbad, CA) following the manufacturer’s instructions, and it was suspended in 30 µl of RNase-free water. DNase I (Takara, Dalian, China) was used to remove the residual genomic DNA. PrimeScript RT Master Mix Perfect Real Time (Takara, Dalian, China) was used to obtain the first-strand cDNA.

qPCR was used for the analysis of the relative expression by iCycler iQ5 (Bio-Rad, Hercules, CA). Three sets of gene-specific primers (QActin-F/QActin-R, QHMGR-F/QHMGR-R and QVG-F/QVG-R) for qPCR were designed for the β-actin, HaHMGR and Vg gene fragments (Table 1). The PCR protocol consisted of 95°C for 30 sec followed by 40 cycles of 95°C for 5 sec and 60°C for 30 sec using SsoFast™ EvaGreen® Supermix (Bio-Rad). The total reaction volume was 20 µl. All reactions were performed in triplicate. The relative expression of HaHMGR mRNA was compared using ANOVA and Tukey’s post-hoc test for multiple comparisons.

### Preparation, Quantification and Purification of dsRNA

The dsRNAs prepared from different sections of the coding region showed the same activity as the full-length dsRNA. A randomly chosen section of the coding region of HaHMGR was amplified from moth cDNA. A control fragment from EGFP was amplified from the pBCMiLR-3xP3 EGFP-PL^−^ vector (kindly provided by Dr. Handler, USDA). A T7 RNA polymerase promoter was added to the EGFP and HaHMGR sequences using PCR by adding the T7 promoter sequence at the 5′ end of the amplification primers (T7EGFP-F/T7EGFP-R or T7HMGR-F/T7HMGR-R) (Table 1). The PCR products were excised from the ethidium bromide-stained gel and purified using a DNA purification kit (Axygen, Hangzhou, China). The dsRNA was synthesized using the T7 RiboMAX™ Express RNAi System (Promega). Samples were incubated at 37°C for 4 h. The nucleic acid was then treated with RQ1 RNase-free DNase (Promega) to remove the DNA template. The dsRNA was purified using the MEGAclear™ Kit (Ambion, Austin, USA). Formation of dsRNAs was confirmed by running 1 µl of these reactions on a 1% agarose gel.

### RNA Interference

A 1,176-bp fragment (dsHaHMGR) and a 520-bp fragment (dsEGFP) were used to generate two different dsRNAs. One microliter of dsHaHMGR (1 µg), 1 µl of dsEGFP (1 µg) or 1 µl of nuclease-free water (blank control) was injected into the abdomen of 2-day-old female pupa. To examine whether silencing of HaHMGR gene influenced ovipostion, injected females were subjected to an oviposition bioassay. Twenty females that were either treated with dsRNA (dsHaHMGR or dsEGFP) or nuclease-free water mated with untreated males in cages containing a 10% sucrose (sugar/water) solution. The cage (40 cm×30 cm×30 cm) experiments were performed in triplicate. The number of eggs laid by each female was recorded.

To further determine the effect of RNA interference on mating, fecundity, and larval emerging, one female treated with dsRNA (dsHaHMGR or dsEGFP) was paired with an untreated male (1∶1) in a small cage (N = 30). Adult females were dissected and examined for the presence of spermatophores to verify mating status under the binocular microscope (Nikon, Japan). The number of eggs and the number hatched larvae were recorded.

### qPCR Analysis of HaHMGR and Vg Expression after RNAi

qPCR was used for detecting HaHMGR and Vg expression levels. qPCR was performed using SsoFast™ EvaGreen® Supermix (Bio-Rad) according to the manufacturer’s instructions on a BioRad iCycler iQ5 (Bio-Rad, Hercules, CA). The assays were performed in triplicate. To avoid the disturbance of off-target effects, the qPCR primers were designed to detect the outside part of the dsRNA fragment. β-actin was chosen as an internal control gene after validation. The relative gene expression data were analyzed using ANOVA and Tukey’s post-hoc test for multiple comparisons.

### Semi-field Trials

To verify the above results in the laboratory, semi-field cage tests were conducted to compare the number of laying eggs and hatched larvae after dsHaHMGR treatment as compared to the control. Twenty pairs of moths were released in a screened quarantine cage (2 m×2 m×2 m) located in a greenhouse, which contained four mature cotton plants. The experiments were performed in triplicate.

## Results

### Cloning of HaHMGR and Vg

A 2,408-bp fragment of the HaHMGR gene was amplified from all individuals and cloned to obtain the full-length cDNA. This gene fragment shared a high homology with *A. ipsilon, B. mori,* and *S. cynthia ricini* HMGR genes in sequence alignment. The 2,408-bp fragment of *H. armigera* showed 83%, 71% and 70% similarity with *A. ipsilon, B. mori,* and *S. cynthia ricini*, respectively. A 187-bp fragment was cloned by the 5′-RACE method, and a 735-bp fragment was cloned by the 3′-RACE method, which allowed identification of the stop codon (TAA). These methods resulted in the cloning of a 3,329-bp full-length cDNA (GenBank accession no. GU584103) of the HaHMGR gene. This cDNA contained a 187-bp 5′-UTR, 2,514-bp ORF, and 628-bp 3′-UTR, and it encoded 837 amino acids.

A 5,129-bp fragment of a presumed Vg homologue of *H. armigera* was obtained by PCR with degenerate primers and cDNA prepared from the total RNA of a female moth as a template. The use of RACE methods allowed a 5,636-bp sequence to be obtained (GenBank accession no. JQ723600), and this sequence encoded a 1,756-aa protein with a predicted molecular mass of 193.2 kDa. A BLAST database search indicated that the protein was the *H. armigera* homologue of Vg. The amino acid sequence was highly conserved showing high percentages of identity to other Vg proteins, including *S. litura* (71%), *B. mori* (56%) and *Nasonia vitripennis* (26%).

### Sequence Alignments and Comparative Analysis

**Figure 1:**
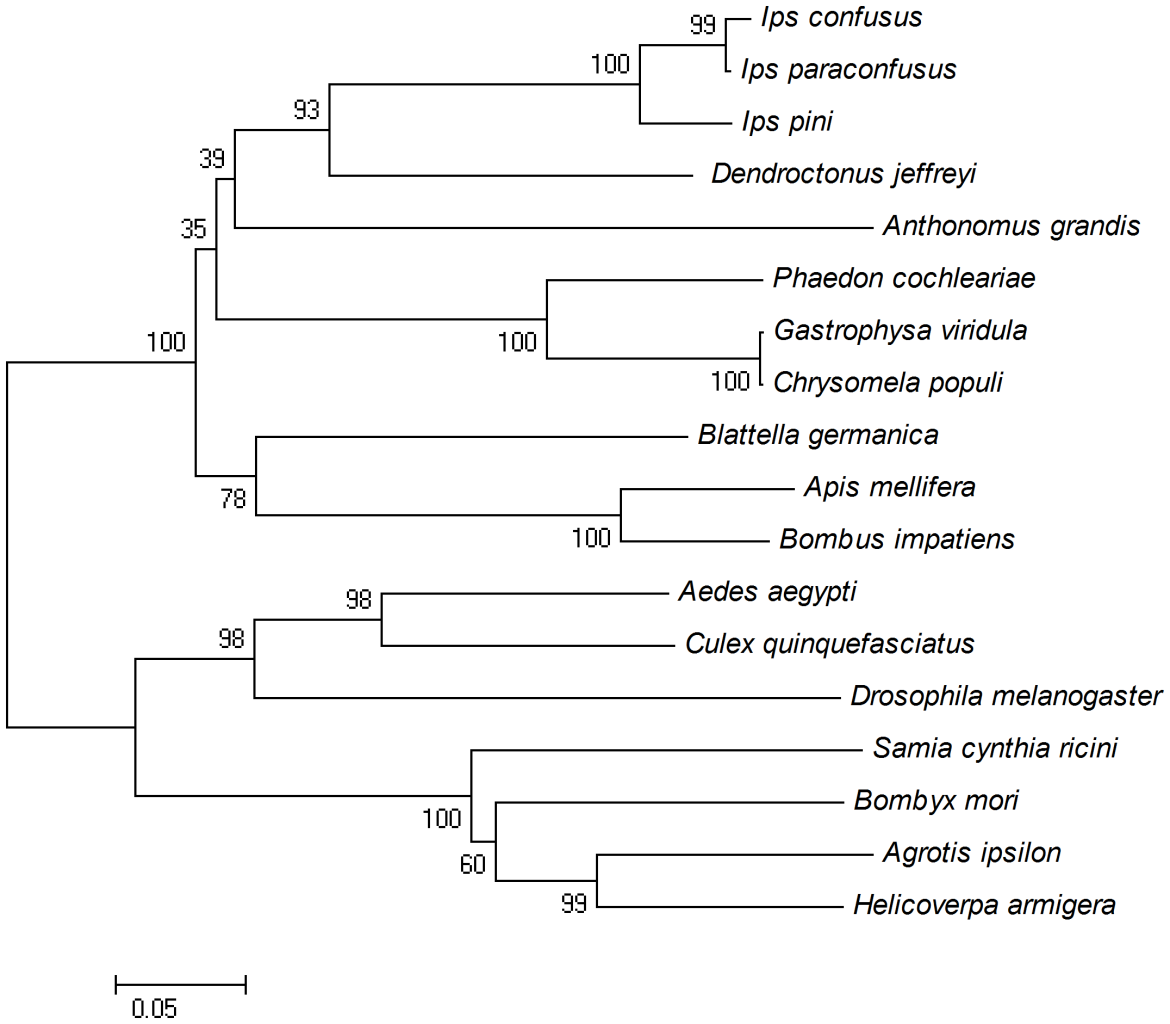
Neighbor-joining phylogenetic tree of the amino acid sequences of HMGR by Molecular Evolutionary Genetics Analysis Software Version 4.0 (MEGA4). The branches were statistically evaluated by bootstrap analysis. All sequences were from GenBank.

The protein sequence of HaHMGR showed 54–98% similarity with other species. The maximal similarity was observed with *A. ipsilon*, which was the closest phylogenetic relative with 95% and 98% similarity for positivity and identity, respectively. Bootstrap values were high in all nodes, and the topology was consistent with the current known phylogenies based on other genes. Regarding the insect species, Lepidoptera and Diptera clustered in phylogenetically coherent groups (Fig. 1).

### Relative Expression of HaHMGR and Vg mRNA in pupae and Effects of RNAi on HaHMGR and Vg Transcription

**Figure 2:**
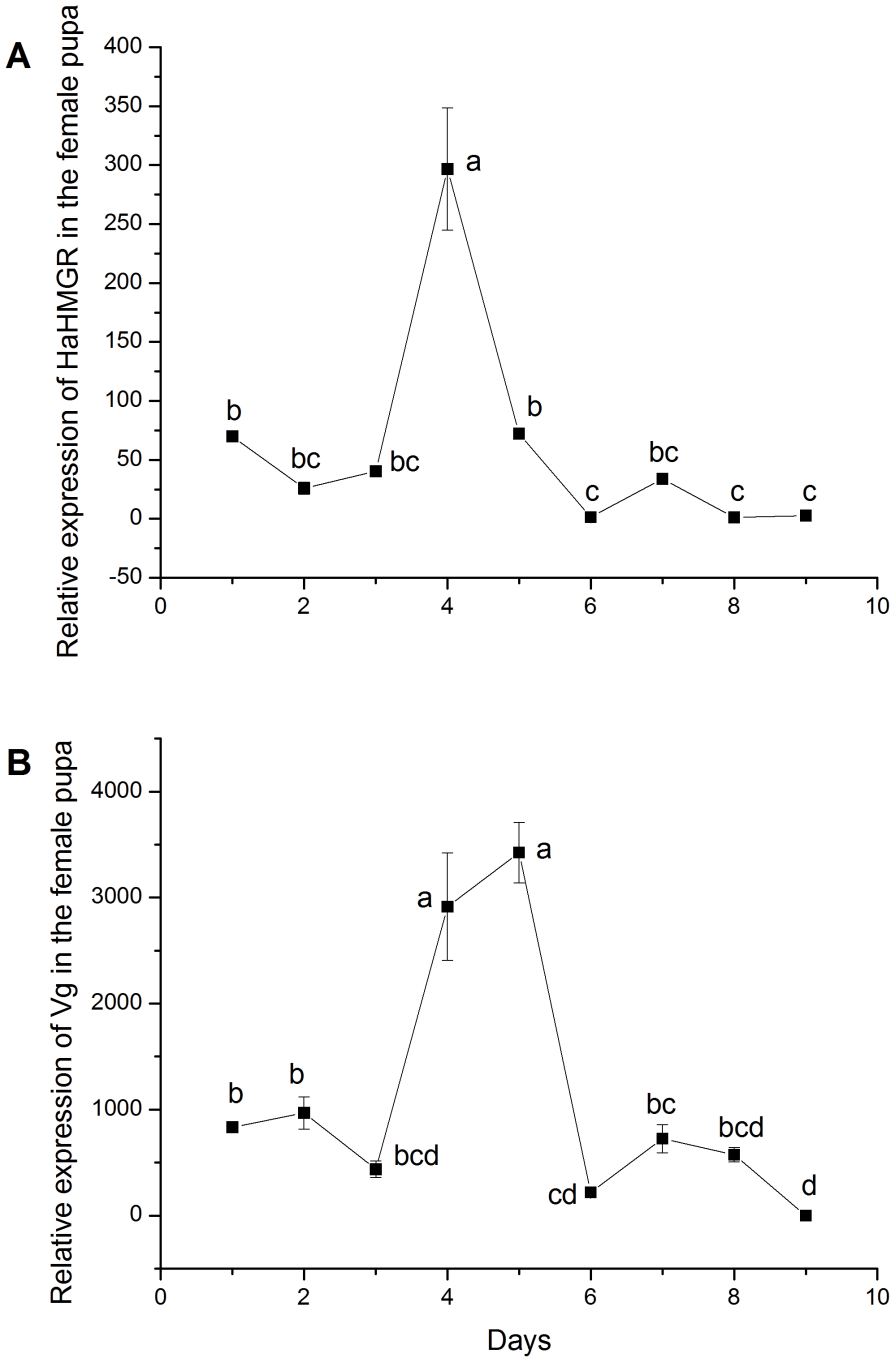
The relative expression of *Helicoverpa armigera* HMGR (HaHMGR) and vitellogenin mRNA in female pupae. (A) The expression pattern of HaHMGR at different ages (day 1 to day 9). (B) The expression pattern of vitellogenin at different ages (day 1 to day 9). The mean and SD values were obtained using SPSS version 16.0. Values with the same letter are not significantly different at the *P*>0.05 level (ANOVA followed by Tukey’s post-hoc test).

The efficiency of qPCR for β-actin, HaHMGR and Vg were qualified (Fig. S1). The relative expression levels of HaHMGR and Vg mRNA in the pupae of *H. armigera* females waved from day 1 to day 9 (Fig. 2). The maximal value for the relative expression of Vg mRNA occurred in the 5-day-old pupa, whereas the peak of the expression of HaHMGR emerged in the 4-day-old pupa.

**Figure 3:**
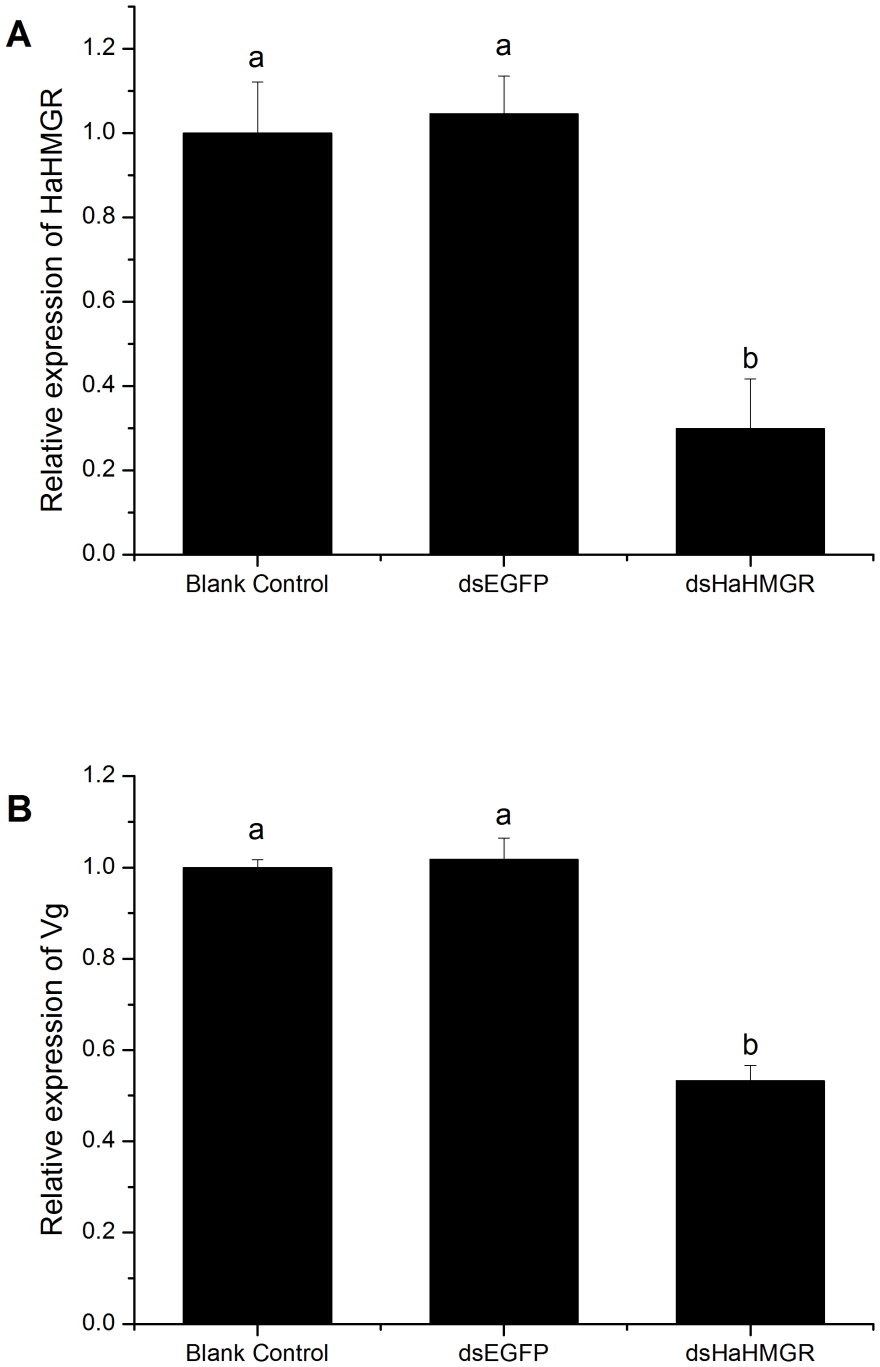
The relative expression of *Helicoverpa armigera* HMGR (HaHMGR) and vitellogenin in *H.* *armigera* after injecting dsRNA. (A) Histograms represent the expression of HaHMGR after injecting HaHMGR double-stranded RNA (dsHaHMGR). (B) The expression of vitellogenin after injecting dsHaHMGR. The enhanced green fluorescent protein double-stranded RNA (dsEGFP) treatment group was used as a negative control, and nuclease-free water was used as a blank control. Values with the same letter are not significantly different at the *P*>0.05 level (ANOVA followed by Tukey’s post-hoc test).

To validate the silencing effects of the RNAi fragments, *H. armigera* gene expression was detected by qPCR. qPCR results showed that the expression levels of HaHMGR from the injected female moths were significantly decreased compared to the controls (Fig. 3A). In addition, the transcription level of Vg mRNA in the injected female moths was also significantly reduced (Fig. 3B). However, there was no difference in the relative expression of HaHMGR or Vg in dsEGFP-injected moths compared to the blank control group.

### Effects of HaHMGR RNAi in *H. armigera*

**Figure 4:**
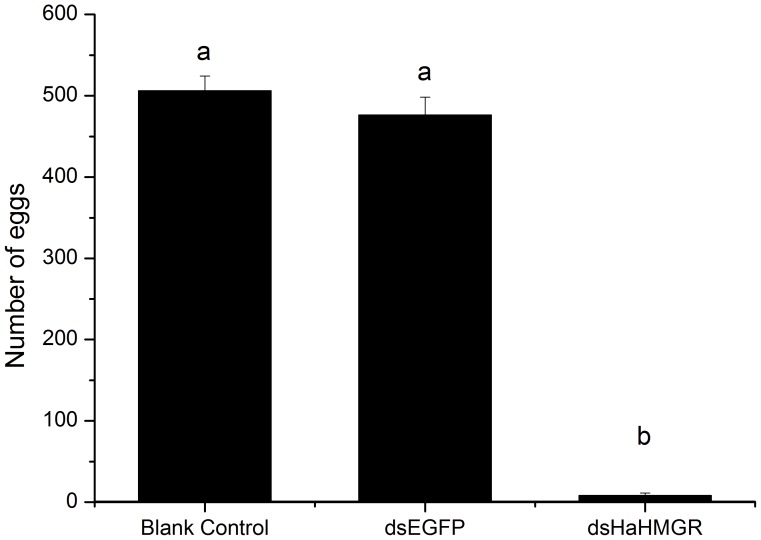
Effects of *Helicoverpa armigera* HMGR (HaHMGR) RNA interference (RNAi) on the oviposition of *H.* *armigera*. Twenty dsRNA-treated or nuclease-free water-treated females were mated with untreated males in cages (40 cm×30 cm×30 cm). The enhanced green fluorescent protein double-stranded RNA (dsEGFP) treatment group was used as a negative control. Nuclease-free water was used as a blank control. Histograms represent the average oviposition per female. Values with the same letter are not significantly different at the *P*>0.05 level (ANOVA followed by Tukey’s post-hoc test).

To study the regulatory role of HaHMGR, the expression of HaHMGR was lowered using systemic RNAi. The eggs laid by dsHaHMGR-, dsEGFP- and blank control-treated females were collected and examined. In the dsHaHMGR-treated group, the egg production of each female was significantly reduced. There was a 98% decrease in egg production when 1 µg of dsHaHMGR was injected into the abdomen of 2-day-old female pupae compared to the blank control. However, there was no effect on egg production for the dsEGFP-treated group and blank control group (Fig. 4). The one-pair experiments demonstrated that both the proportion of valid mating and fecundity were zero. No spermatophores were observed in the dsHaHMGR-treated group (Table 2).

**Table 2:** Effect of dsHaHMGR on fecundity, larval production and number of spermatophores in *Helicoverpa armigera*.

	blank control	dsEGFP-treated (negative control)	dsHaHMGR-treated
Proportion of valid mating	53.33%	46.67%	0
Fecundity	1137.63±241.50a	1113.00±141.29a	0b
Number of larvae emerging	493.63±212.90a	422.00±56.79a	0b
Proportion of larvae emerging	0.43±0.13a	0.37±0.08a	-
Number of spermatophores	1.71±0.71a	2.20±0.90a	0b

Two-day-old female pupae were treated with 1 µg of HaHMGR double-stranded RNA (dsHaHMGR) or enhanced green fluorescent protein double-stranded RNA (dsEGFP) (negative control). One female treated with dsRNA (dsHaHMGR or dsEGFP) was paired with an untreated male in a small cage (N = 30). Values are expressed in absolute terms as a percentage or as the mean ± SD. Values with the same letter are not significantly different at the *P*>0.05 level (ANOVA followed by Tukey’s post-hoc test).

### Semi-field Trials

The semi-field cage experiments showed the same results in the dsHaHMGR-treated group, which was consistent with the results in the laboratory. Oviposition was decreased by approximately 99% after dsHaHMGR treatment in a cage located in a greenhouse (Table 3). There were no hatched larvae in the cotton plants after dsHaHMGR treatment.

**Table 3:** Fecundity and number of emerging *Helicoverpa armigera* larvae in the greenhouse cage trial.

	dsEGFP-treated (negative control)	dsHaHMGR-treated
Fecundity	901.00±184.01a	8.67±4.04b
Number of larvae emerging	152.67±39.70a	0b

Twenty pairs of moths were released in a screened quarantine cage (2 m×2 m×2 m) located in a greenhouse, which contained four mature cotton plants. The total number of eggs or larvae was recorded. Values are expressed in absolute terms as the mean ± SD. Values with the same letter are not significantly different at the *P*>0.05 level (ANOVA followed by a *t-*test).

## Discussion

In the present study, HaHMGR was cloned and characterized as the HMGR homologue from the cotton bollworm, *H. armigera*. This is the first report that used RNA interference to demonstrate the regulatory role of the HaHMGR gene on the oviposition of *H. armigera*. The results clearly suggested that silencing of the HaHMGR gene influenced the fecundity of the females and effectively reduced the oviposition in *H. armigera*. Silencing the HaHMGR gene also decreased the levels of vitellogenin mRNA expression.

The HaHMGR gene showed the characteristic genetic organization of HMGR enzymes, which was confirmed by the following criteria: (i) the cDNA yielded an amino acid sequence showing 98%, 90% and 88% homology with *A. ipsilon*, *B. mori* and *S. cynthia ricini* sequences, respectively [22], [23], [24]; (ii) the hydrophobicity plot of the protein (from cDNA) showed classical organization of animal-type HMGR with a N-terminal region containing the potential membrane-spanning domains followed by a short linker that connects the C-terminal region containing the catalytic domain [29]; (iii) eight membrane-spanning domains were present in the hydrophobicity plot, which was consistent with other animal-type HMGR sequences [30]; and (iv) the catalytic domain in the C-terminal region included *His809*, which is known to be a conserved region in all HMGR sequences characterized to date [31].

Importantly, the relative levels of HaHMGR mRNA exhibited a significant increase in 4-day-old female pupae. Based on this result, 2-day-old female pupae were treated by injecting dsRNA of the target gene to determine the efficiency of RNAi in moths at this developmental stage.

Because RNAi is a knockdown method, silencing is not complete, and the effect is transient [32]. The life stage of insects is one of the important factors that influences the silencing effect [4]. We conducted experiments to choose the appropriate life stage in this moth for silencing the target gene. For example, no silencing effect was observed in the adult moth after treating 5^th^ instar larvae of *H. armigera* with HaHMGR dsRNA by injection (1 µg) or feeding in the larval diets (10 µg) (unpublished results). A previous report has also shown that feeding long dsRNA to *H. armigera* larvae is not successful [33]. In contrast, a strong silencing effect was observed in female moths after injecting dsRNA into 2-day-old female pupae.

The present results suggested that HaHMGR RNAi treatment effectively inhibited oviposition in *H. armigera* compared to the control. HMGR was the first gene to be cloned in the mevalonate pathway [28], and it is also the most widely studied gene in this pathway in insects [24]–[28], [34], [35] because of its potential regulatory role in the production of JH in insects. It is likely that the knockdown of HaHMGR decreases the biosynthesis of JH and then reduces the expression of vitellogenins. In response to the suppression of JH, the mevalonate pathway can also produce other final products, such as dolichol, which behaves as a donor of oligosaccharide residues in the glycosylation of proteins, e.g., in the synthesis of vitellogenins in most insect species. In this case, HaHMGR RNAi may also result in suppression of the production of dolichol, thereby further affecting oogenesis. Importantly, no valid mating of the dsHaHMGR-treated group occurred in the laboratory one-pair experiments. A previous study has reported that compactin (HMGR inhibitor) clearly inhibits sex pheromone biosynthesis in the silkworm (*B. mori*) and the common cutworm (*S. litura*) [36]. The lack of valid mating of the dsHaHMGR-treated group may have resulted from the decreased biosynthesis of sex pheromone in *H. armigera*. Further studies are needed to investigate the common mechanisms underlying the basal regulation role of HMGR in insects for potential pest control applications using RNAi.

The larvae of *H. armigera* feed on a wide range of economically important crops and cause a tremendous loss in yield. Although transgenic Bt plants have proven to be successful in controlling this pest, sporadic cases of development of resistance against Bt toxin may jeopardize this accomplishment [37]. Therefore, it is necessary to develop alternate and novel strategies to control this pest. The present results demonstrated that the HaHMGR gene can be a potential target for effective insect control. Releasing female moths that have been molecularly sterilized by silencing the HaHMGR gene shows considerable promise against this pest and other similar insect pests. In the present study, semi-field trials in cages demonstrated great success for controlling *H. armigera* by demonstrating their failure to produce fertile progeny. In addition, the currently available data suggests that insect-resistant transgenic plants generated using plant RNAi-mediated silencing of the insect HaHMGR gene may also represent a novel strategy against insect pests.

## Supporting Information

Figure S1 **The qPCR efficiency data graphs for β-actin,** ***Helicoverpa armigera*** **HMGR (HaHMGR) and vitellogenin.** We made 10 times diluted concentration gradient for the standard sample. Three repeats for each concentration gradient. (A) The standard curve of the β-actin in qPCR. The amplification efficiency was 103.66%. (B) The standard curve of the HaHMGR in qPCR. The amplification efficiency was 95.93%. (C) The standard curve of the vitellogenin in qPCR. The amplification efficiency was 98.34%.
(TIF)
