# Supplementary figures and images for: RNAi Silencing of the HaHMG-CoA Reductase Gene Inhibits Oviposition in the Helicoverpa armigera Cotton Bollworm
Source: PLoS One. 2013 Jul 2;8(7):e67732. doi: 10.1371/journal.pone.0067732 (PMC3699641; doi:10.1371/journal.pone.0067732)

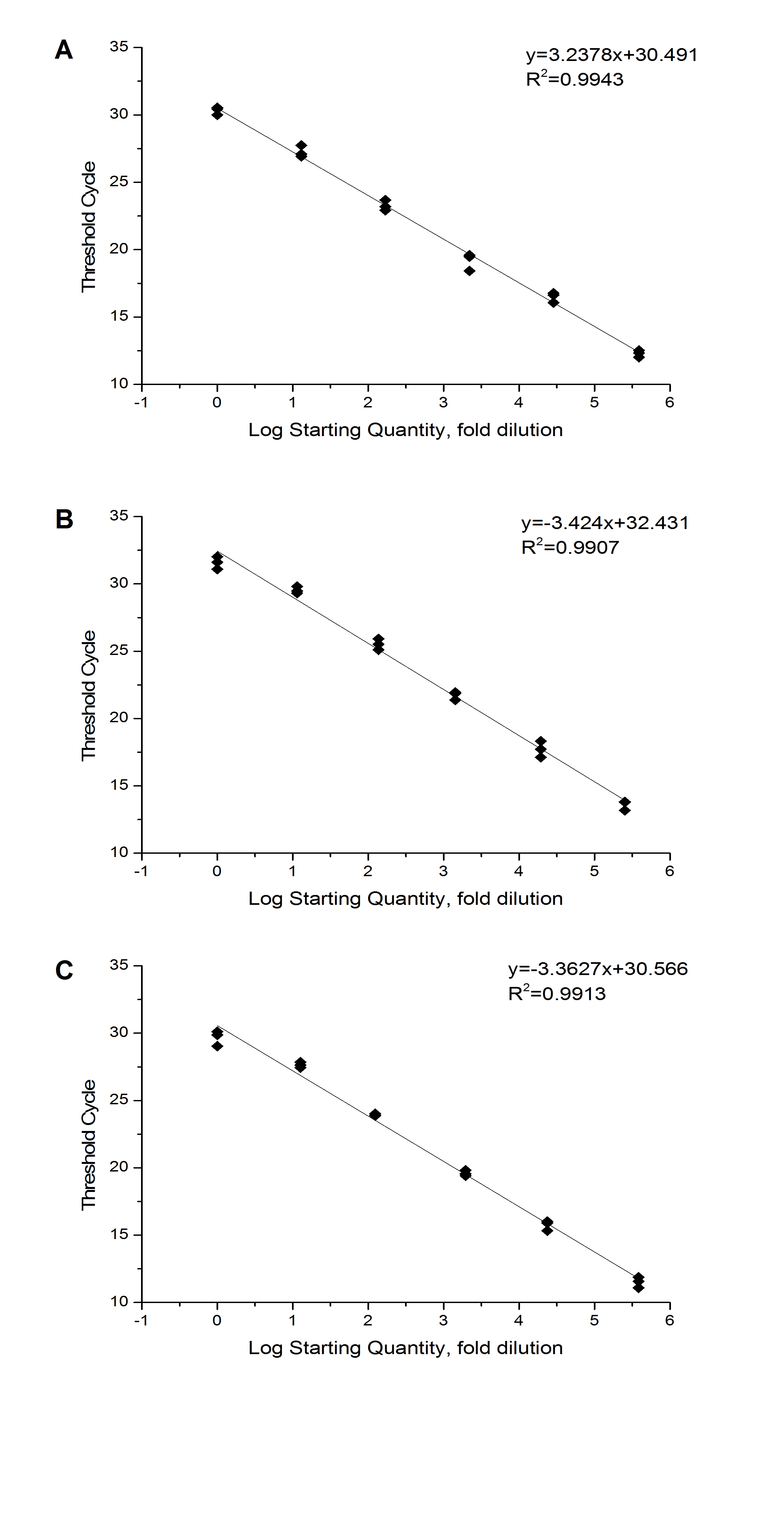

Supplement: Figure S1 — The qPCR efficiency data graphs for β-actin, Helicoverpa armigera HMGR (HaHMGR) and vitellogenin. We made 10 times diluted concentration gradient for the standard sample. Three repeats for each concentration gradient. (A) The standard curve of the β-actin in qPCR. The amplification efficiency was 103.66%. (B) The standard curve of the HaHMGR in qPCR. The amplification efficiency was 95.93%. (C) The standard curve of the vitellogenin in qPCR. The amplification efficiency was 98.34%. (TIF) [file pone.0067732.s001.tif]
